# Supplementary material for: The interactome of intact mitochondria by cross-linking mass spectrometry provides evidence for coexisting respiratory supercomplexes
Source: Mol Cell Proteomics. 2017 Dec 8;17(2):216–32. doi: 10.1074/mcp.RA117.000470 (PMC5795388; doi:10.1074/mcp.RA117.000470)
Supplement: Supplemental Data [file supp_RA117.000470_133922_0_supp_23978_fzffwf.zip › spectra_annotation/mito_spectra_annotation/179-1-17-1-34-1.pdf]

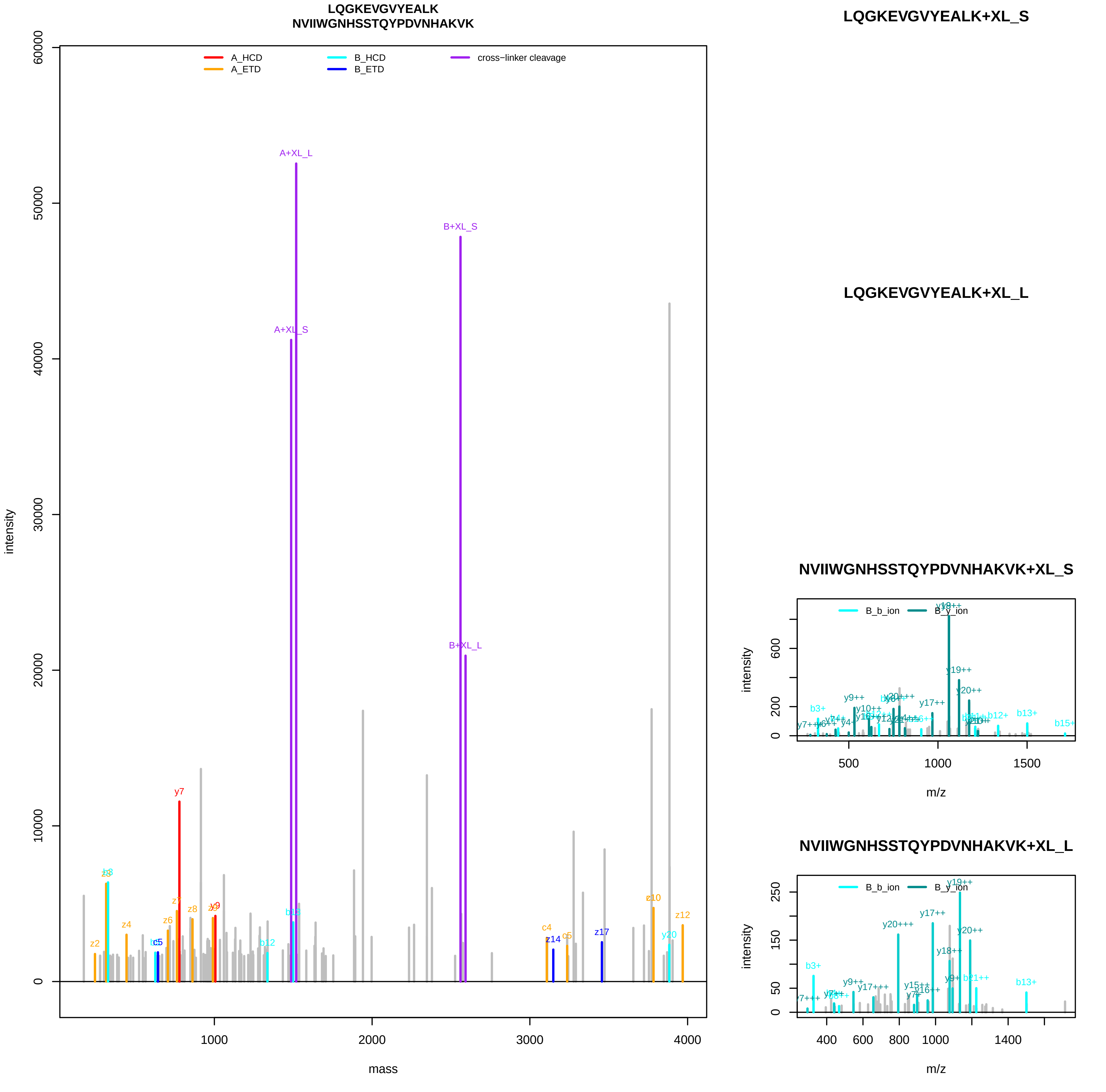

LQGKEVGVYEALK
NVIIWGNHSSTQYPDVNHAKVK
A_HCD
A_ETD
B_HCD
B_ETD
cross−linker cleavage
intensity
mass
A+XL_L
A+XL_S
B+XL_S
B+XL_L
y7
y9
z2
z3
z4
z6
z7
z8
z9
b3
b5
c5
b12
b13
c4
z14
z17
z10
z12
y20
0
10000
20000
30000
40000
50000
60000
1000
2000
3000
4000
LQGKEVGVYEALK+XL_S
LQGKEVGVYEALK+XL_L
NVIIWGNHSSTQYPDVNHAKVK+XL_S
B_b_ion
B_y_ion
y19++
y20++
y17++
y9++
y10++
y20+++
b3+
b12+
b13+
b15+
m/z
500
1000
1500
200
600
NVIIWGNHSSTQYPDVNHAKVK+XL_L
y18++
y17+++
y15++
y16++
b21++
400
800
1400
50
150
250
